# Utility of Dexrazoxane for the Attenuation of Epirubicin-Induced Genetic Alterations in Mouse Germ Cells

**DOI:** 10.1371/journal.pone.0163703

**Published:** 2016-09-30

**Authors:** Sabry M. Attia, Sheikh F. Ahmad, Mushtaq A. Ansaria, Ahmed Nadeem, Othman A. Al-Shabanah, Mohammed M. Al-Harbi, Saleh A. Bakheet

**Affiliations:** 1 Pharmacology and Toxicology Department, Faculty of Pharmacy, King Saud University, 11451, Riyadh, Saudi Arabia; 2 Pharmacology and Toxicology Department, Faculty of Pharmacy, Al-Azhar University, Nasr City, Cairo, Egypt; Northwestern University Feinberg School of Medicine, UNITED STATES

## Abstract

Dexrazoxane has been approved to treat anthracycline-induced cardiomyopathy and extravasation. However, the effect of dexrazoxane on epirubicin-induced genetic alterations in germ cells has not yet been reported. Thus, the aim of this study was to determine whether dexrazoxane modulates epirubicin-induced genetic damage in the germ cells of male mice. Our results show that dexrazoxane was not genotoxic at the tested doses. Furthermore, it protected mouse germ cells against epirubicin-induced genetic alterations as detected by the reduction in disomic and diploid sperm, spermatogonial chromosomal aberrations, and abnormal sperm heads. The attenuating effect of dexrazoxane was greater at higher dose, indicating a dose-dependent effect. Moreover, sperm motility and count were ameliorated by dexrazoxane pretreatment. Epirubicin induced marked biochemical changes characteristic of oxidative DNA damage including elevated 8-hydroxy-2ʹ-deoxyguanosine levels and reduction in reduced glutathione. Pretreatment of mice with dexrazoxane before epirubicin challenge restored these altered endpoints. We conclude that dexrazoxane may efficiently mitigate the epirubicin insult in male germ cells, and prevent the enhanced risk of abnormal reproductive outcomes and associated health risks. Thus, pretreating patients with dexrazoxane prior to epirubicin may efficiently preserve not only sperm quality but also prevent the transmission of genetic damage to future generations.

## Introduction

Anthracyclines are highly effective against mesenchymal malignancies and epithelial tumors [1]. They include numerous chemotherapeutic agents, for example doxorubicin and its analog epirubicin (4ʹ-epidoxorubicin), which differs from doxorubicin in the steric position of the 4ʹ-OH group. Epirubicin is generally used because it has an equivalent antitumor spectrum of action to that of doxorubicin but shows lesser cardiac and systemic toxicity than doxorubicin [2]. Similar to other anthracyclines, epirubicin acts by intercalating DNA strands, which induces complex formation and further prevents RNA and DNA synthesis. Epirubicin also triggers DNA cleavage by DNA topoisomerase II, thereby activating mechanisms that lead to cell damage. In addition, epirubicin produces free radicals that cause DNA and cell damage [3].

Similar to other anti-cancer agents, high doses of anthracyclines induce programmed cell death [3]. Furthermore, several lines of evidence show that low doses of anticancer agents induce mitotic catastrophe resulting from abnormal mitotic events of improper chromosomal separation and cell division [4]. Epirubicin has been reported to cause genetic damage in animals and humans [5–8]. Such damage can give rise to secondary malignances in somatic cells. However, when germ cells are affected, the genetic damage can not only affect sperm quality but can also induce genetic hazards to future generations [9]. Numerical chromosomal aberrations such as diploidy and disomy in gametes are the most common causes of fetal loss and fertility impairment in humans [10, 11]. In newborns, numerical chromosomal aberrations are a frequent cause of congenital defects and mental retardation such as Down’s syndrome. Moreover, more than 35% of spontaneous abortions are due to numerical chromosomal aberrations, and the most common cases are those with XO constitution and trisomies of chromosomes 16, 21, and 22 [12]. Syndromes involving additional or missing sex chromosomes carry fewer severe morphological defects but are associated with drastically impaired fertility [13].

Dexrazoxane is approved to protect the skin and heart from anthracycline-induced extravasation and cardiotoxicity [14, 15]. Cells cleave dexrazoxane during regular metabolism, generating an ethylenediaminetetraacetic acid derivative that chelates iron to decrease oxidative stress, thereby activating the cellular mechanism that protects the heart from acute anthracycline-induced injury [16, 17]. Importantly, dexrazoxane has not been shown to inhibit the antitumor effects of anthracyclines in pediatric patients with leukemia, or those patients with breast cancer, and does not increase the risk for development of secondary tumors [18, 19]. A possible explanation for this apparent contradiction was provided in a set of mechanistic experiments by Yan *et al*. [20], which suggested that dexrazoxane protected normal but not tumor cells. This study demonstrated that while dexrazoxane blocked anthracycline-induced double-stranded DNA breaks in HTETOP cells, it did not inhibit doxorubicin-induced programmed cell death that occurred *via* reduced glutathione (GSH) depletion in a topoisomerase II-independent manner [20].

Despite the intensive use of dexrazoxane in epirubicin-induced extravasation and heart failure, no information is available in the literature on its potential to induce genetic damage in germ cells. Therefore, evaluation of the effects of co-treatment with dexrazoxane and epirubicin on genetic alterations in the germ cells is important to obtain more insights into the genetic alterations induced by epirubicin that may play a role in the occurrence of the reproductive toxicity. In this study, a series of related but distinct cytogenetic techniques were used, including sperm fluorescence *in situ* hybridization (FISH) method and spermatogonial metaphase chromosomal analysis, to determine genetic damage in the germ cells. Epididymal sperm analysis after exposure to dexrazoxane, epirubicin, or both was performed to assess the influence of this combination on sperm quality. Moreover, the total testis and epididymal sperm levels of GSH and DNA 8-hydroxy-2ʹ-deoxyguanosine (8-OHdG), indicators of DNA oxidative stress, were measured as a possible mechanism underlying this mitigation.

## Materials and Methods

### Animals

Adult male Swiss albino mice, aged 9–11 weeks and weighing 18–24 g were acquired from the animal house, Faculty of Pharmacy, King Saud University, Riyadh, Kingdom of Saudi Arabia. Mice were kept in our smaller animal house for one week under standard conditions of humidity, light (12 h light/dark cycle), and temperature (24 ± 3°C). Mice were fed standard mice chow and water *ad libitum*. All procedures conducted with the mice were in accordance with the standards in the guidelines for the care and use of experimental animals. The study protocol was approved by the Animal Ethics Committee of the Faculty of Pharmacy, King Saud University. The vehicle control and treatment groups consisted of five randomly assigned mice each.

### Drugs and treatment

**Fig 1:**
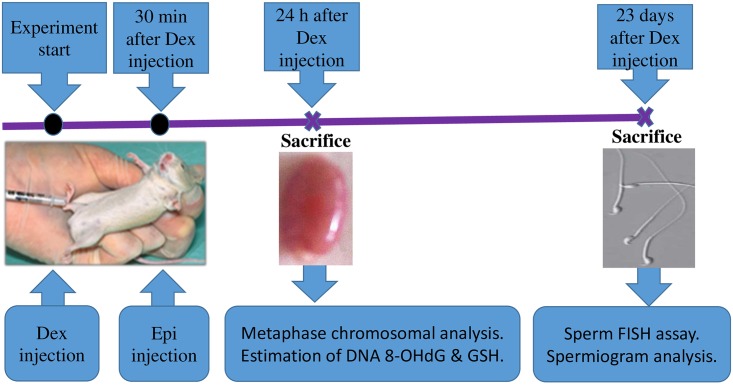
Schematic overview of drug treatments, sampling times, and evaluated parameters. Dex = dexrazoxane and Epi = epirubicin.

Epirubicin and dexrazoxane, which were obtained from Sigma-Aldrich (St. Louis, MO, USA), were dissolved in dimethyl sulfoxide (DMSO, 10%) in normal saline and were injected intraperitoneally within 1 h following preparation. The experimental design of the epirubicin and dexrazoxane treatment, sampling times, and the assessed parameters are schematically shown in Fig 1. Epirubicin was injected at doses of 3–12 mg/kg, which were selected based on previous clastogenic and aneugenic studies in mice [6, 7]. Dexrazoxane at 75 and 150 mg/kg has previously shown promising protection against genomic damage induced by podophyllotoxins in mice [21–23] and epirubicin-induced genetic damage in mice bone marrow cells [24]. Furthermore, the selected doses are within the chemotherapeutic dose range for humans. The control mice were injected with the same amounts of DMSO in normal saline.

### Sperm FISH assay

The sperm FISH assay was used to determine the frequency of aneuploidy during meiotic divisions of spermatocytes. Animals were euthanized by cervical dislocation 23 days after the injection of epirubicin, dexrazoxane, or both, and then sperm were isolated from the *cauda epididymis*. Sperm sampling time was based on the published timeline of the 5-bromo-2'-deoxyuridine (BrdU)-incorporation assay for epirubicin [7]. The BrdU-incorporation assay data demonstrate that epirubicin induced a significant prolongation of meiotic divisions in mouse spermatocytes [7]. In the epididymal sperm analysis, a crucial prerequisite for the timing of sperm sampling is a precise knowledge of both duration of meiotic stages and possible temporal changes of meiotic divisions induced by the chemical aneugen. Schmid *et al*. [25] suggest that the influence of a test chemical on meiotic cell cycle progression should be determined before designing sampling time of sperm cells in order to optimize the test protocol and to avoid missing an effect by inappropriate timing of the sperm samples.

**Fig 2:**
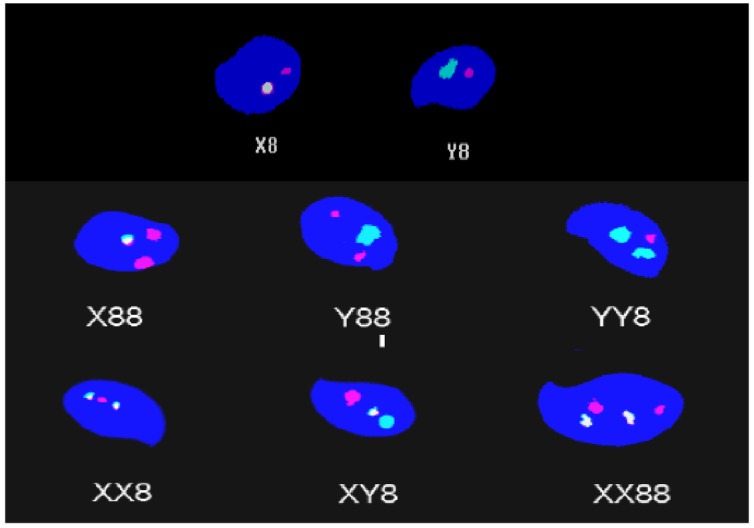
Representative images of various signal combinations in epididymal sperm. X8 and Y8, XY8 to X88, and XX88 are normal, disomic, and diploid sperm, respectively. Sperm were hybridized with DNA probes specific for chromosomes X (yellow signal with fluorescein isothiocyanate (FITC and CY3), Y (green signal with FITC), and 8 (red signal with CY3).

The frequencies of diploid and disomic sperm cells were assessed by sperm FISH assay with DNA probes specific for mouse chromosomes 8, Y, and X, in which each probe was labeled with a different color. The hybridization, washing, and amplification of the signals were carried out as described previously [26]. The sperm cells were examined for aneuploidy using a Nikon fluorescence microscope. Fluorescence signals from coded slides were scored in approximately 10000 sperm cells per animal, and the sperm cells were classified as normal (Y8 and X8), diploid (XX88, YY88, and XY88), or disomic (XY8, X88, and Y88) (Fig 2).

### Spermatogonial metaphase chromosomal analysis

In the spermatogonial metaphase chromosomal analysis that was performed 20 h post-treatment with epirubicin, dexrazoxane, or both, the mice were intraperitoneally treated with 4 mg/kg colchicine. The mice were euthanized 4 h later, the testes separated, and then the slides prepared as described previously [27]. Briefly, the separated testes were washed with 2.2% tri-sodium citrate, and then the seminiferous tubules were teased-out and soaked in 1% tri-sodium citrate solution at room temperature for 20 min. The solution was removed completely, and the tubules were suspended in Carnoy’s fixative (methanol/glacial acetic acid, 3 + 1). The fixative was changed three times and the tubules were then dispersed with Pasteur pipettes in 50% glacial acetic acid to obtain spermatogonia. After centrifugation, the pellets were suspended in fresh fixative and 2–3 drops of the cell suspension were put on a microscopic slide, which had been wetted with 70% ethanol and flame dried. The slides were then stained with 5% Giemsa and had cover slips affixed using Eukitt mounting medium.

**Fig 3:**
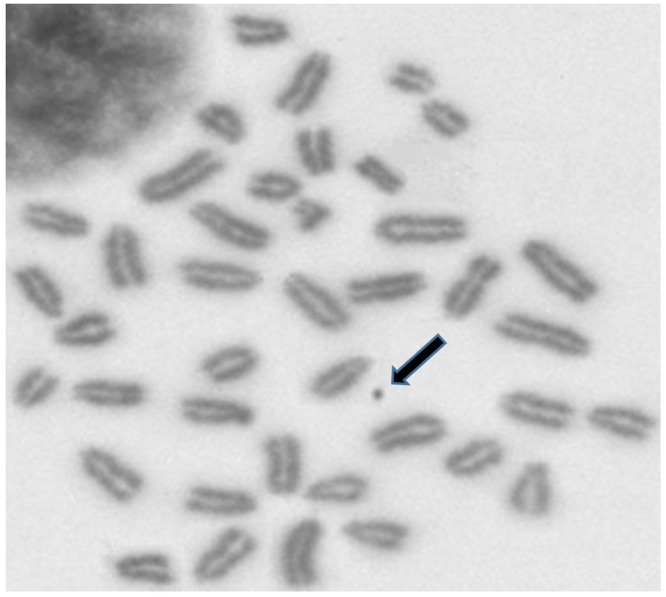
Spermatogonial metaphase with a structural chromosomal aberration: fragment (arrow).

Strict scoring criteria were used to distinguish chromosome complements to avoid misinterpreting the data. First, slides were screened for regions of suitable technical quality, where the metaphase chromosomes were well spread. The types of aberrations recorded included chromatid/chromosome breaks, chromatid/chromosome gaps, and fragments [27]. Breaks were true discontinuities with clearly dislocated fragments. They were distinguished from achromatic lesions (gaps), which do not represent true discontinuities in the DNA. It is generally assumed that gaps are sites of despiralisation in the metaphase chromosome, which render the DNA invisible under light microscopy. It has been proposed that an achromatic lesion may actually be a single-strand break in the DNA double helix because of incomplete excision repair and thus may represent a point of possible instability [28]. Therefore, gaps are always noted but reported separately from true chromosomal aberrations. Approximately 500 well-spread spermatogonial metaphases from each group were investigated, and the percentage of total structural chromosomal aberrations was recorded. The average aberrations/100 spermatogonial metaphases, excluding gaps (due to their controversial genetic significance), were calculated. Fig 3 shows an example of spermatogonial metaphase with a structural chromosomal aberration.

### Epididymal sperm analysis

**Fig 4:**
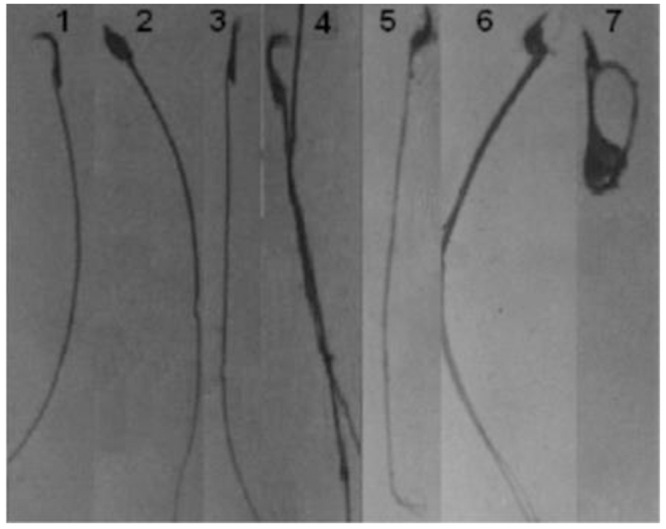
Representative images of epididymal sperm showing normal sperm (1), amorphous head (2), hookless (3), banana-shaped (4) and abnormal tails (5–7).

Sperm cells from the same animals that were euthanized for the sperm FISH assay were also evaluated for sperm parameters. Specifically, total sperm number and motility were determined using a bright field microscope with a Neubauer-hemocytometer according to the World Health Organization (WHO) guide as previously described [29]. For sperm morphology, aliquots of sperm suspensions were stained with eosin-Y (1%), smeared on glass slides, air-dried, and fixed to make them permanent. Coded slides were then examined by a light microscope, and the abnormal morphologies were classified as closely to those previously described [30]. About 500 sperm cells per mouse were evaluated for morphological abnormalities such as banana-shaped, hookless, amorphous, and tail abnormalities (Fig 4).

### Measurement of 8-OHdG and GSH levels

To study the influence of dexrazoxane on DNA oxidative damage induced by epirubicin, two separate experiments were performed. In the first experiment, mice were euthanized 24 h after treatment with epirubicin (with or without dexrazoxane pretreatment) and their testes isolated. In the second experiment, mice were euthanized 23 days after treatment with epirubicin (with or without dexrazoxane pretreatment) and their sperm cells isolated. In both experiments, testicular and sperm DNA were isolated using DNA Extractor WB (Wako Chemicals, USA), the isolated DNA was digested with DNase-1, and then 8-OHdG levels were measured using the Bioxytech 8-OHdG enzyme-linked immunosorbent assay kit (ELISA) kit (OXIS Health Products) as described previously [31]. The stated values were the means of triplicate measurements, and 8-OHdG levels are presented as ng/μg DNA. Additionally, the testicular and sperm GSH levels were assessed based on the reduction of Ellman's reagent [5,5΄-dithiobis (2-nitrobenzoic acid)] by the thiol groups of GSH to produce 2-nitro-S-mercaptobenzoic acid [32]. The product was measured spectrophotometrically at 412 nm. Protein estimation was performed based on the technique of Lowry *et al*. [33] using bovine serum albumin (BSA) as a standard.

### Data analysis

The data were analyzed using the Mann-Whitney *U* and Kruskal-Wallis tests followed by Dunn’s multiple comparison test. A *P* = 0.05 was selected as the criterion for significance.

## Results

### Sperm FISH assay

The patterns of fluorescent signals in epididymal sperm visualized by the sperm FISH assay and examples of normal, five classes of disomic sperm and one class of diploid sperm are presented in Fig 2. The results of the sperm FISH assay are shown in Table 1. Dexrazoxane did not significantly increase the frequencies of disomic and diploid sperm at any of the doses tested. A significant increase in diploid and disomic sperm was induced by treatment with the two highest doses of epirubicin compared to the control value. The dose-response curves for epirubicin-induced disomic and diploid sperm can be described by the linear equations y = 0.021x + 0.029 (r^2^ = 0.99) and y = 0.0144x + 0.015 (r^2^ = 0.93), respectively. In contrast, dexrazoxane pre-treatment significantly reduced epirubicin-induced diploid and disomic sperm, and the higher dose of dexrazoxane induced greater amelioration (*P*<0.01).

**Table 1:** Sperm fluorescence *in situ* hybridization (FISH) of epididymal sperm from animals treated with indicated doses of dexrazoxane (Dex), epirubicin (Epi), or both.

Treatment (mg/kg)	% Disomies	% Diploidies
Control	0.052 ± 0.014	0.004 ± 0.005
Dex (75)	0.060 ± 0.014	0.004 ± 0.005
Dex (150)	0.062 ± 0.013	0.006 ± 0.005
Epi (3)	0.068 ± 0.017	0.008 ± 0.004
Epi (6)	0.092 ± 0.022*	0.026 ± 0.008**
Epi (12)	0.114 ± 0.018**	0.046 ± 0.011**
Dex (75) + Epi (3)	0.062 ± 0.004	0.008 ± 0.004
Dex (75) + Epi (6)	0.066 ± 0.025	0.018 ± 0.008
Dex (75) + Epi (12)	0.082 ± 0.014^a^	0.020 ± 0.007^a^
Dex (150) + Epi (3)	0.054 ± 0.011	0.006 ± 0.005
Dex (150) + Epi (6)	0.058 ± 0.010^b^	0.012 ± 0.008^b^
Dex (150) + Epi (12)	0.066 ± 0.011^a^	0.014 ± 0.008^a^

The frequencies of disomic and diploid sperm cells were assessed by DNA probes specific for the mouse sex chromosomes X and Y as well as the mouse chromosome 8 (an example of autosomal chromosomes and available at our laboratory) each labeled with a different color. Values are mean ± standard deviation (SD).

*P < 0.05

**P < 0.01 compared with vehicle control group (Kruskal-Wallis test followed by Dunn’s multiple comparisons test).

^a^*P* < 0.05

^b^*P* < 0.01 compared with corresponding group treated with epirubicin alone (Mann-Whitney *U*-test).

### Spermatogonial metaphase chromosomal analysis

**Fig 5:**
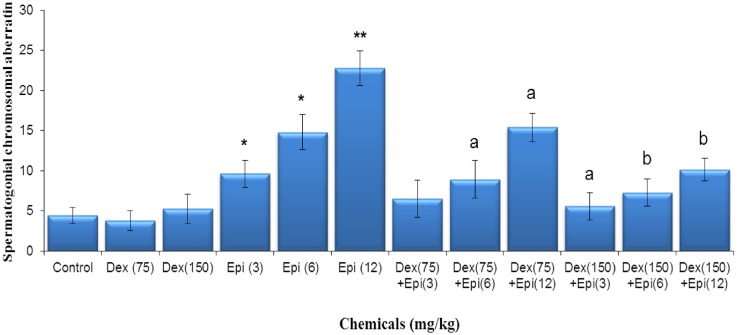
Frequency of spermatogonial chromosomal aberrations induced by indicated doses of dexrazoxane (Dex), epirubicin (Epi), or both. Spermatogonial chromosomal aberration was expressed as mean ± standard deviation (SD) of total aberrations/100 spermatogonial metaphases. *P < 0.05 and **P < 0.01 compared to control (Kruskal-Wallis test followed by Dunn’s multiple comparisons test). ^a^*P* < 0.05 and ^b^*P* < 0.01 compared to epirubicin alone (Mann-Whitney *U*-test).

An example of spermatogonial metaphase with a structural chromosomal aberration is given in Fig 3. The results of spermatogonial metaphase chromosomal analysis (Fig 5) revealed that dexrazoxane alone did not affect spermatogonial chromosomes, confirming its lack of clastogenicity. On the other hand, epirubicin treatment increased the frequencies of spermatogonial chromosomal aberrations in a dose-dependent manner. The dose-response curve for epirubicin-induced spermatogonial chromosomal aberrations can be described by the linear equation y = 6.0186x−2.129 (r^2^ = 0.98). Conversely, pretreatment of mice with dexrazoxane prior to epirubicin injection significantly decreased the rates of metaphase chromosomal aberrations compared to those aberrations induced by epirubicin alone, and while the higher dose of dexrazoxane showed greater protection (*P*<0.01).

### Epididymal sperm analysis

Representative microscopic images of normal sperm and various abnormal shapes in epididymal sperm of mice are presented in Fig 4. As shown in Table 2, dexrazoxane treatment did not affect the parameters of sperm quality that were examined in this study. The fast moving sperm cells dramatically decreased in epirubicin-treated mice compared to that in vehicle control mice. In concert, the percentage of immotile sperm cells significantly increased in epirubicin-treated mice compared to that in vehicle control (*P*<0.01). Furthermore, sperm count significantly decreased in mice injected with epirubicin alone (*P*<0.01). Conversely, the changes in total sperm count and motility were reversed following dexrazoxane pretreatment. After epirubicin treatment, normal sperm morphology significantly decreased while total spermatozoa abnormalities significantly increased as compared to control animals (*P*<0.01). On the other hand, pretreatment of animals with dexrazoxane significantly reduced the incidence of abnormal sperm cells compared to epirubicin exposure alone (*P*<0.05).

**Table 2:** Epididymal sperm analysis of animals treated with indicated doses of dexrazoxane (Dex), epirubicin (Epi), or both.

Groups and chemicals (mg/kg)	Motility (%)			Morphology (%)			Sperm counts (10^6^ /ml)
	Fast	Slow	Immotile	Normal	Abnormal heads	Abnormal tails	
Control	64.2 ± 8.75	12.2 ± 3.03	23.2 ± 6.34	92.6 ± 2.02	4.00 ± 0.70	3.3 ± 1.55	62.2 ± 7.36
Dex (150)	60.6 ± 9.31	13.0 ± 2.91	26.4 ± 7.63	89.0 ± 4.06	5.86 ± 2.10	5.1 ± 2.01	54.4 ± 9.35
Epi (12)	34.8 ± 9.65*	9.6 ± 2.30	55.6 ± 11.9**	78.6 ± 6.3**	10.18 ± 2.7**	12.1 ± 3.4**	29.2 ± 5.3**
Dex (150) + Epi (12)	51.2 ± 10.7	13.4 ± 3.50	35.4 ± 11.1^a^	87.6 ± 4.0^a^	5.6 ± 2.0^a^	6.6 ± 2.4^a^	54.6 ± 6.6^b^

Values are mean ± standard deviation (SD).

*P < 0.05

**P < 0.01 compared with vehicle control group (Kruskal-Wallis test followed by Dunn’s multiple comparison test).

^a^*P* < 0.05

^b^*P* < 0.01 compared with group treated with epirubicin alone (Mann-Whitney *U*-test).

### 8-OHdG and GSH levels

**Fig 6:**
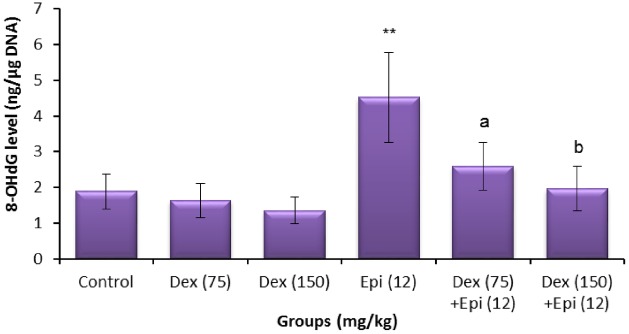
Testicular levels of 8-hydroxy-2ʹ-deoxyguanosine (8-OHdG) of animals treated with dexrazoxane (Dex), epirubicin (Epi), or both. Values are mean ± standard deviation (SD). **P < 0.01 compared with vehicle control group (Kruskal-Wallis test followed by Dunn’s multiple comparison test). ^a^*P* < 0.05 and ^b^*P* < 0.01 compared with group treated with epirubicin alone (Mann-Whitney *U*-test).

**Fig 7:**
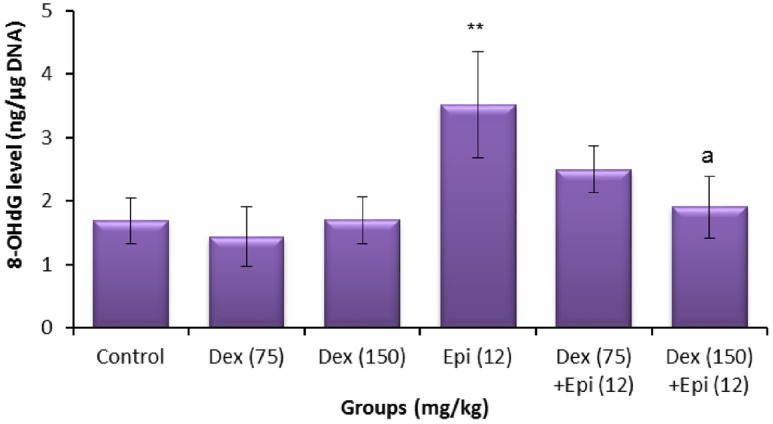
Sperm levels of 8-hydroxy-2ʹ-deoxyguanosine (8-OHdG) of animals treated with dexrazoxane (Dex), epirubicin (Epi), or both. Values are mean ± standard deviation (SD). **P < 0.01 compared with vehicle control group (Kruskal-Wallis test followed by Dunn’s multiple comparison test). ^a^*P* < 0.05 compared with group treated with epirubicin alone (Mann-Whitney *U*-test).

**Fig 8:**
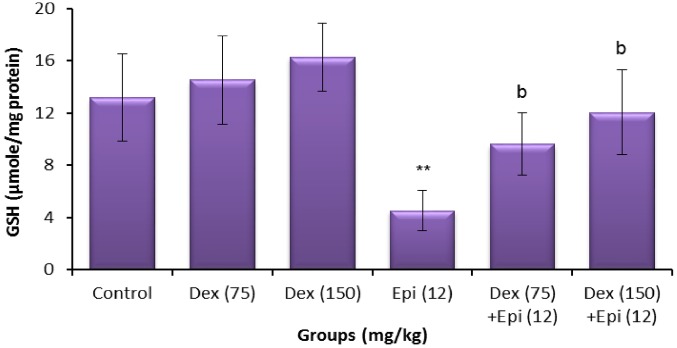
Testicular glutathione (GSH) levels of animals treated with dexrazoxane (Dex), epirubicin (Epi), or both. Values are mean ± standard deviation (SD). **P < 0.01 compared with vehicle control group (Kruskal-Wallis test followed by Dunn’s multiple comparison test). ^b^*P* < 0.01 compared with group treated with epirubicin alone (Mann-Whitney *U*-test).

**Fig 9:**
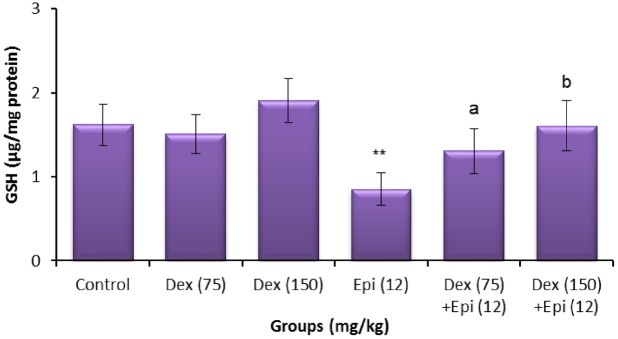
Sperm glutathione (GSH) levels of animals treated with dexrazoxane (Dex), epirubicin (Epi), or both. Values are mean ± standard deviation (SD). **P < 0.01 compared with vehicle control group (Kruskal-Wallis test followed by Dunn’s multiple comparison test). ^a^*P* < 0.05 and ^b^*P* < 0.01 compared with group treated with epirubicin alone (Mann-Whitney *U*-test).

The results of testicular and sperm estimation of 8-OHdG and GSH are presented in Figs 6–9. As shown in Figs 6 and 7, treatment with dexrazoxane failed to induce any significant change in the levels of testicular and sperm 8-OHdG at the tested doses. A significant increase in testicular and sperm 8-OHdG was observed in the epirubicin-treated mice compared to the solvent control groups (*P*<0.01). With regard to the animals treated with dexrazoxane before epirubicin, a weak protection in testis only was observed with 75 mg/kg of dexrazoxane (Fig 6). With 150 mg/kg pre-treatment, however, dexrazoxane produced a clear significant suppressive effect on the testicular and sperm 8-OHdG induced by epirubicin in comparison to the epirubicin alone (Figs 6 and 7).

As shown in Figs 8 and 9, there was no significant difference in the level of testicular and sperm GSH between the dexrazoxane-treated and control mice at any of the doses tested. The testicular and sperm GSH level observed in epirubicin-treated mice was significantly decreased as compared to the control value (*P*<0.01). In contrast, the epirubicin-induced decrease in testicular GSH was significantly increased in dexrazoxane-pretreated mice compared to the reported value with epirubicin treatment alone (Fig 8). Furthermore, mice pretreated with dexrazoxane showed a significant increase in sperm level of GSH compared to epirubicin-treated mice (Fig 9). In addition, the values significantly differed from that of the testicular and sperm GSH after treatment with epirubicin alone and the higher dose of dexrazoxane induced greater improvement (*P*<0.01).

## Discussion

Dexrazoxane is approved to treat cardiomyopathy and extravasation caused by anthracyclines [14, 15]. However, the effect of dexrazoxane on epirubicin-induced genetic alterations in germ cells has not yet been reported. Thus, the aim of this work was to ascertain whether co-administration of dexrazoxane would mitigate the epirubicin-induced genetic alterations in male germ cells. This study showed that dexrazoxane was not genotoxic at the tested doses. Furthermore, it protected the mouse germ cells against epirubicin-induced genetic alterations as detected by reduction in disomic and diploid sperm, spermatogonial chromosomal aberrations, and sperm head abnormality. Total sperm number and motility were also restored following dexrazoxane pretreatment. In the present work, we attempted to elucidate the possible mechanisms underlying the anti-genotoxic action of dexrazoxane by examining its inhibition of free radical production. The data revealed that free radical production by epirubicin was significantly inhibited by pretreatment with dexrazoxane.

Human studies have demonstrated that several chemotherapy regimens increase the incidence of genetic alterations in germ cells, indicating that cancer patients undergoing treatment with these agents may be at a higher risk for abnormal reproductive outcomes [34]. Thus, it is of pertinent importance that male cancer patients be made aware that certain treatment regimens could result in diminished sperm quality or quantity due to an increase in genetic alterations in spermatogenic cells. In this study, epirubicin caused a dose-dependent increase in the percentage of diploid and disomic sperm cells, and the effect was significant at the two highest tested doses. Pretreatment of the animals with dexrazoxane significantly reduced epirubicin-induced diploid and disomic sperm and the higher dose induced greater protection. The results of our *in vivo* study on the effects of epirubicin treatment were in agreement with results of an earlier *in vitro* study with Chinese hamster cell cultures that showed epirubicin increased the hypodiploidy and hyperdiploidy of the cells [35]. Additionally, histological and flow cytometric analyses of mouse spermatogenesis displayed an increase in the coefficient of variation in the DNA histogram, a measure of aneuploidy, and an increase of diploid spermatids after epirubicin exposure [8].

Chromosomal aberrations are a significant event in tumorigenesis. A threshold level of DNA damage may exist that leads to an overload of the DNA repair mechanisms, and a significant increase in aberrant metaphases. In the present study, average chromosomal aberrations/100 metaphases in the vehicle control group was 4.5%, and this value corresponded well with that previously reported by Palo *et al*. [36] with the same age and strain of mice. Dexrazoxane alone failed to increase chromosomal aberrations in spermatogonial cells confirming its lack of clastogenicity. However, a significant increase in incidences of spermatogonial chromosomal aberrations was caused by treatment with all investigated doses of epirubicin. These findings were in concordance with previous observations that epirubicin induced structural chromosomal aberrations in both humans and animals [6, 37, 38]. In contrast, pretreatment of animals with dexrazoxane prior to the epirubicin injection significantly decreased the rates of clastogenic changes compared to epirubicin alone in a dose-dependent manner.

Disturbances in the reproductive system are a common adverse effect of chemotherapeutic agents [9, 39]. Treated male patients usually show poor sperm quality with increased abnormal sperm morphology and decreased total sperm number and motility. This high percentage of immotile or poorly motile sperm may not induce fertilization [40]. In the current study, dexrazoxane treatment did not affect the studied sperm quality parameters. On the other hand, percentages of sperm motility and count significantly decreased in epirubicin-treated mice. Moreover, epirubicin significantly increased the frequency of abnormal sperm while pretreatment with dexrazoxane ameliorated the decreased percentage of sperm count and motility. Additionally, the frequency of abnormal sperm was restored following dexrazoxane pretreatment. The decreased sperm quality after treatment with epirubicin was consistent with our previous results that exposure to epirubicin significantly increased aneuploid sperm [7]. Therefore, these findings are in agreement with previous results that patients with poor sperm quality show increased sperm diploidy and disomy rates [41].

The precise mechanisms by which dexrazoxane protects against epirubicin-induced genetic alterations in germ cells are not fully understood. A possible explanation for this protection is that concurrent treatment with dexrazoxane scavenged free radicals produced by epirubicin before they could affect the DNA and cause genetic damage. In fact, the metabolism of epirubicin is known to produce free radicals [42]. These free radicals may damage biological molecules including DNA and protein, eventually inducing cell damage, genetic alterations, and secondary tumors [43]. It has been reported that chromosomal aberrations and DNA damage in germ cells are closely related to male infertility and 8-OHdG is a sensitive biomarker of oxidative DNA damage caused by generation of free radicals in human germ cells [44]. In this study, to determine whether the anti-genotoxic effects of dexrazoxane were due to an improvement in scavenging of free radicals produced after exposure to epirubicin, GSH and 8-OHdG levels were assessed after the mice were injected with epirubicin, dexrazoxane, or both.

The results revealed that dexrazoxane pretreatment significantly decreased the epirubicin-induced testicular and sperm 8-OHdG levels, and inhibited the reduction in the testicular and sperm GSH. The improved GSH levels suggest that the protection by dexrazoxane may be mediated by modulation of cellular antioxidant levels. This finding is in concordance with previous experiments where dexrazoxane was reported to scavenge hydroxyl free radicals [45]. Moreover, dexrazoxane has a stronger intrinsic scavenging activity than typical antioxidants such as GSH, against not only hydroxyl free radicals, the typical free-radical product of redox reaction of iron complexes, but also peroxynitrite and peroxyl radicals. These findings demonstrate that the antioxidant effects of dexrazoxane are not solely dependent on iron chelation, although the latter may contribute to decreased iron-based free radical production [45, 46]. Consequently, the antioxidant property of dexrazoxane might be responsible for mitigating the genetic alterations induced by epirubicin in the germ cells.

## Conclusion

The current study revealed that dexrazoxane was not genotoxic at the doses tested. Moreover, it may provide an efficient way to mitigate epirubicin-induced genetic damage in germ cells and reduce the associated risk for abnormal reproductive outcomes without decreasing epirubicin’s therapeutic efficiency, as demonstrated by its clinical effectiveness in averting both epirubicin-induced extravasation and cardiotoxicity. Performing *in vitro* fertilization studies that could track the rate of fertilization and development to blastocyst would be interesting and might provide more insights into the protective effect of dexrazoxane against reproductive toxicity induced by anthracyclines, and will be considered as a focus for our future study.
