# Correction: Utility of Dexrazoxane for the Attenuation of Epirubicin-Induced Genetic Alterations in Mouse Germ Cells

**DOI:** 10.1371/journal.pone.0165854

**Published:** 2016-10-27

**Authors:** Sabry M. Attia, Sheikh F. Ahmad, Mushtaq A. Ansari, Ahmed Nadeem, Othman A. Al-Shabanah, Mohammed M. Al-Harbi, Saleh A. Bakheet

The third author's name is spelled incorrectly. The correct name is: Mushtaq A. Ansari. The correct citation is: Attia SM, Ahmad SF, Ansari MA, Nadeem A, Al-Shabanah OA, Al-Harbi MM, et al. (2016) Utility of Dexrazoxane for the Attenuation of Epirubicin-Induced Genetic Alterations in Mouse Germ Cells. PLoS ONE 11(9): e0163703. doi:10.1371/journal.pone.0163703
